# Surgical clip closure of the left atrial appendage

**DOI:** 10.1111/jce.15181

**Published:** 2021-08-04

**Authors:** Fabrizio Rosati, Gijs E. de Maat, Mattia A. E. Valente, Massimo A. Mariani, Stefano Benussi

**Affiliations:** ^1^ Division of Cardiac Surgery Spedali Civili Brescia University Hospital Brescia Italy; ^2^ Department of Cardio‐thoracic Surgery University Medical Centre Groningen Groningen The Netherlands

**Keywords:** ablation, atrial fibrillation, left atrial appendage

## Abstract

Atrial fibrillation (AF) is the most common atrial arrhythmia, but it is not a benign disease. AF is an important risk factor for thromboembolic events, causing significant morbidity and mortality. The left atrial appendage (LAA) plays an important role in thrombus formation, but the ideal management of the LAA remains a topic of debate. The increasing popularity of surgical epicardial ablation and hybrid endoepicardial ablation approaches, especially in patients with a more advanced diseased substrate, has increased interest in epicardial LAA management. Minimally invasive treatment options for the LAA offer a unique opportunity to close the LAA with a clip device. This review highlights morphologic, electrophysiologic, and surgical aspects of the LAA with regard to AF surgery, and aims to illustrate the importance of surgical clip closure of the LAA.

## 1 INTRODUCTION

Atrial fibrillation (AF) is the most common atrial arrhythmia. It should not be considered a benign disease, in addition to symptom burden, loss of atrial and potentially ventricular function, AF is an important risk factor for cerebral stoke. This causes significant morbidity and mortality, especially in the aging populations of the developed countries. The left atrial appendage (LAA) plays an important role in thrombus formation. Data suggest that approximately 90% of atrial thrombi in nonrheumatic AF are found within the LAA.^1^ Although oral anticoagulants (OACs) offer an important reduction in stroke risk, they also increase bleeding risk.

Benefits of rhythm control for more persistent forms of AF are becoming clearer, but results of catheter ablation remain far from ideal.^2^ This led to a growing interest in minimally invasive AF surgery,^3^, ^4^, ^5^, ^6^ especially for patients who are refractory to medical and transcatheter therapy.^4^, ^7^, ^8^

To date, transcatheter closure of the LAA shows no significant benefit compared to OAC. During thoracoscopic AF ablation, the surgeon has the unique opportunity to manage the LAA. In the past, complete LAA exclusion has proven challenging, while incomplete closure or amputation may increase thrombo‐embolic risk.^9^, ^10^ However, clip occlusion devices have simplified surgical LAA management with consistent exclusion and, importantly, electrical isolation of the LAA.^11^, ^12^ Since the introduction of epicardial LAA management, over 250,000 clip occlusions have been performed in patients worldwide. A recent large randomized controlled trial showed a significant reduction in ischemic stroke when LAA amputation or closure was performed as a concomitant procedure, in AF patient undergoing cardiac surgery,^13^ demonstrating the potential benefit of appendage exclusion. This review highlights morphologic, electrophysiologic, and surgical aspects of the LAA with regard to AF surgery, and aims to illustrate the practical implications for surgical rhythm management.

## 2 LAA MANAGEMENT AND EFFECT ON LA AND LV MECHANICAL FUNCTION

Historically considered a useless embryonic remnant,^14^ the importance of the LAA is increasingly being recognized. Not merely its role as “the most lethal human attachment”^15^ due to its role in cardiac embolisms in AF patients, but also the important role in plays for both LA and LV functions. The LAA can act as a complementary reservoir that refills during left ventricular early systole when the mitral valve is closed and the LA is passively filled. The LAA has been shown to have greater compliance than the LA, particularly in diseases causing volume overload. Therefore, the LAA can play an important role in cardiac pressure‐volume regulation.^1^ However, decreased LA reservoir function is noted after LAA exclusion without affecting intrinsic LA contractility.^16^ During early diastole, the LAA should be considered a compliance chamber that passively fills the LA and LV once the mitral valve is open, while during late diastole, the LAA acts as booster pump due to its intrinsic contractile function, thus contributing to stroke volume.^17^, ^18^ The LAA is calculated to contribute to up to 10% of the total LA volume.^19^ However, contemporary series seem to exclude significant echocardiographic variation in LV stroke volume or LVEF in patients in sinus rhythm undergoing LAA occlusion.^16^ These data have been confirmed in patients with reduced LVEF (<35%),^20^, ^21^, ^22^ Despite a global increase in LA reservoir volume, a moderate improvement in conduit and booster pump function may be expected based on the Frank‐Starling mechanism. It has to be noted that these findings were from endocardial LAA occlusion; little is known about hemodynamic effects of epicardial LAA occlusion.

## 3 LAA MANAGEMENT AND NEUROENDOCRINE EFFECTS

The endocrine role of the LAA is related to the presence of intrinsic stretch‐sensitive receptors able to release ANP. Experimental analysis has demonstrated the presence of so‐called “ANP densely granulated cells” with the highest concentration in the LAA.^23^, ^24^, ^25^ Fluid infusion at the level of the LAA increases blood ANP levels, leading to increased heart rate, natriuresis and diuresis, resulting in reduction of volume load, vasodilatation, and blood pressure decrease.^26^ Several studies demonstrated significant downregulation and inhibition of the natriuresis pathway when the LAA is excluded surgically. Furthermore, noradrenaline, adrenaline, renin, and aldosterone are significantly downregulated in patients treated with epicardial LAA exclusion.^27^, ^28^, ^29^ The appendage is richly innervated by sympathetic and parasympathetic nerves in strict relation to stretch receptors, and interconnected with the angiotensin system activated by ANP release. Turagam et al.^30^ demonstrated a significant reduction of systolic and diastolic blood pressure in hypertensive AF patients undergoing epicardial ablation and LAA exclusion, underlining the necessity to reduce antihypertensive medications. Because the original Cox‐Maze III operation incorporated excision of the LAA down to its base as well as excision of the right atrial appendage tip, patients developed severe postoperative fluid overload due to the decrease of ANP levels in the blood.^31^ Spironolactone should be administered postoperatively to counter this effect. The period of spironolactone administration is at least 1 month in our clinical practice.

## 4 LAA MORPHOLOGY

**Figure 1:**
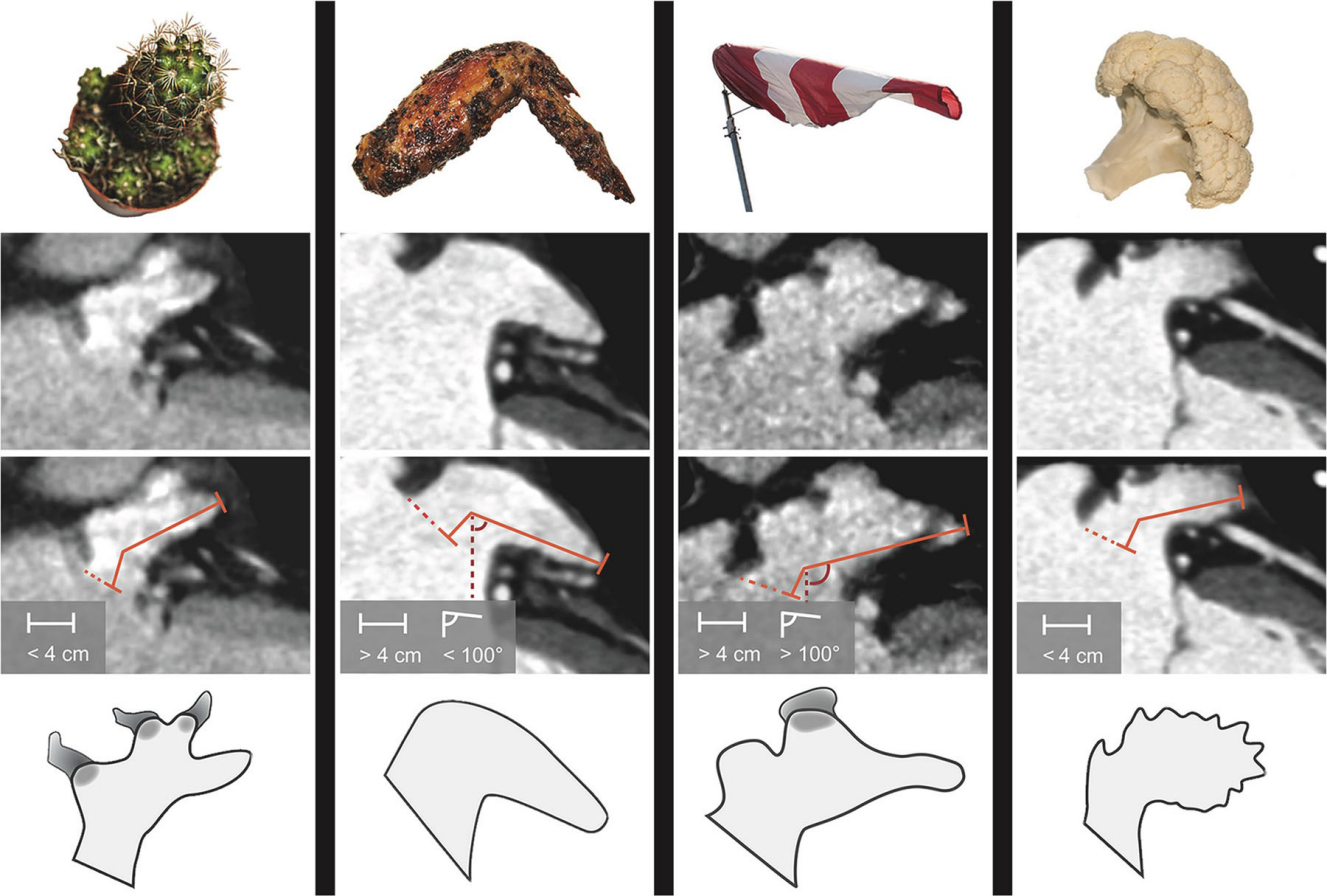
Wang's classification for LAA morphologies. LAA length was measured from the orifice area (dashed orange line) to the farthest point of the LAA via the center of the main lobe. The bend angle was measured with an imaginary vertical line (red dashed line) and a line between the main lobe and the farthest point of the LAA. Cactus has a dominant central lobe, one or more secondary lobes, and total length <4 cm. ChickenWing has only one lobe, total length more than 4 cm, and a bend angle less than 100°. WindSock has one dominant lobe with several secondary, or even tertiary lobes, total length more than 4 cm, and a bend angle of over 100°. CauliFlower has a total length less than 4 cm and complex internal structures. LAA, left atrial appendage

Increased interest in ablation techniques and management of the LAA, combined with improved imaging modalities, have led to classification systems for LAA morphology. The most commonly used Wang classification assigns the LAA to “Cactus,” “Chicken wing,” “Windsock,” or “Cauliflower” (Figure 1)^32^ morphologies. Such classifications system may assist both in planning interventions and the identification of “dangerous” appendages that require intervention.^33^ Large LAAs and LAAs with single lobe morphology are more frequently present in patients who suffer from stroke. However, AF‐induced elevated pressure and subsequently increased LAA volume may alter LAA morphology. The number of LAA lobes is reduced due to the remarkably thin (+/−1 mm) wall, combined with the lack of supporting tissue around the appendage. Additionally, bending of the LAA may occur due to dilatation, creating turbulence and eventually increasing thrombo‐embolic risk.

This knowledge may guide patient selection for LAA management,^34^ although more studies on this subject are required. For procedural planning, other imaging modalities such as contrast enhanced cardiac computerized tomography (CT) can be utilized, although the static nature of CT means differentiation between thrombus and sluggish flow can be difficult, as images are captured a few seconds after contrast reaches the left heart (LA/LAA). Cardiac magnetic resonance imaging is a promising modality for reliable assessment of LAA shape and dimensions/volume. At times, CCT and CMR can be challenging since irregular heart rate and tachycardia significantly reduce image quality (with an ideal heart rate <65 beats/min). Transesophageal echocardiography (TOE) is used to assess the preprocedural presence of thrombus in the LAA.

## 5 THE ROLE OF LAA CLIP OCCLUSION IN THROMBOEMBOLIC STROKE RISK REDUCTION

In general, the LAA is the most common site for cardiac thrombi in patients, both in AF and in SR.^35^ LA enlargement promotes stasis and thus thromboembolic risk.^36^ An additional stroke risk is the presence of a heavily trabeculated LAA endocardium.^33^

Although there is a strong relationship between AF and stroke, the causal relationship between stroke and thrombus formation within the LAA is not undisputed. It has been demonstrated that in AF patient population, 91% of all thrombi were formed within the LAA.^37^ In another prospective study, TEE examination show that of 230 patients with AF for >2 days, 33 out of 34 cardiac thrombi were located in the LAA.^38^ As a part of the original Cox‐Maze procedure, the atrial appendices were routinely amputated, and his group has published extensively on rhythm outcome but also stroke reduction benefit after Maze surgery.^39^ The recent LAAOS III trial, randomizing AF patients undergoing cardiac surgery to concomitant LAA closure or not, demonstrated that LAA closure results in a relative stroke risk reduction of 33% at 3.8 years follow‐up.^13^ Besides stroke risk reduction interventions, patients may present with a thrombus whithin the LAA. A small case series demonstrated that V‐shaped clip can safely exclude partially thrombosed LAAs with minimal manipulation.^40^, ^41^

## 6 THE ROLE OF LAA CLIP OCCLUSION IN RHYTHM CONTROL

Remodeling of the LAA may contribute to the pathogenesis of atrial arrhythmias.^42^ Electrical exclusion of the LAA therefore provides an antiarrhythmogenic benefit. Di Biase et al.^43^ reported that 27% of their AF ablation patient population had foci in the LAA, while the LAA was the only mapped trigger in 8.7% of cases. In transcatheter ablations studies in AF, arrhythmia‐free survival was better in patients who undergo additional LAA isolation compared to pulmonary vein isolation alone; Heeger et al.^44^ showed 49% arrhythmia free survival compared to 37% in the control group (*p* = 0.02). Similar results were achieved in the BELIEF Trial.^45^ Nevertheless, there is concern about potential excess stroke risk after electrical isolation of the LAA via catheter ablation, since the procedure creates an akinetic cul‐de‐sac in communication with the left atrium, which has proven to be highly thrombogenic.^45^ Epicardial LAA clip exclusion offers a potential solution to this problem. Using the clip to occlude the LAA, complete electrical isolation of the LAA has been demonstrated, excluding potential AF onset triggers.^46^ In the long term, the LAA will regress completely following clip placement. However, there are currently no clear data available demonstrating the additional benefit of LAA clip closure as an addition to thoracoscopic PVI, and more studies are warranted. In rare cases of LAA‐driven automatic arrhythmias, stand‐alone epicardial clip LAA closure is quite consistently an effective rhythm control strategy and—not infrequently—the best chance for permanent treatment of the arrhythmia.^12^

## 7 INDICATIONS FOR EPICARDIAL LAA CLIP CLOSURE

The first line treatment to prevent thromboembolism is of course anticoagulation. In recent years, non‐vitamin K oral anticoagulants (NOAC) have proven to be safe and give pharmacological advantages over vitamin K antagonists due to more predictable pharmacokinetics and less need for regular bloodwork and monitoring.^47^

Current guidelines for AF management suggest considering LAA occlusion as a stroke prevention strategy in patients who have experienced cerebral hemorrhage or other bleeding complications related to long‐term anticoagulation therapy and/or patients with AF undergoing cardiac surgery (Class IIb, Level of evidence B).^48^ These indications are based on randomized clinical trials demonstrating noninferiority of percutaneous devices versus anticoagulation therapy in stroke reduction for patients with AF, creating a basis for LAA closure as an alternative to oral anticoagulation.^49^, ^50^, ^51^ However, there is a lack of data providing specific comparisons between percutaneous versus surgical occlusion devices and/or epicardial LAA closure versus NOAC for stroke prevention. The results of the LAAOS III trial, as described previously, could translate to a Class I recommendation for concomitant LAA closure in AF patients with CHA2DS2‐VASc ≥2 undergoing cardiac surgery. Accurate detection of underlying cardiomyopathies should be pursued and the benefits of LAA occlusion (thromboembolic prophylaxis and rhythm control), should be balanced against the potential for hemodynamic impairment, especially in patients without persistent arrhythmias. Patients with AF and end‐stage renal failure (clearance <15 ml/min) have a contraindication for NOAC but also high risk of bleeding; epicardial LAA occlusion in this specific subset of patients is still undefined, but might offer a clinical benefit. Whether or not to exclude the LAA should be discussed in a multidisciplinary approach by the AF‐Heart Team^52^: a case‐by‐case analysis of clinical indications, anatomical and morphological considerations. Utilization of risk scores is warranted to provide a patient‐tailored approach (guided by CHA2DS2‐VASc and HASBLED scores).

In addition to the previously mentioned indications, the 2020 EHRA/EAPCI expert consensus statement on catheter based LAA appendage occlusion summarizes indications for LAA occlusion^53^ as follows: (1) AF patients eligible for long‐term OAC/NOAC who refuse medical treatment despite thorough explanation. (2) AF patients with an absolute contraindication for long‐term OAC/NOAC (hemorrage or side‐effects due to VKA/NOAC). (3) AF patients not compliant to medical therapy.

According to the experts, in case of contraindication to antiplatelet therapy, patients may not be eligible for endovascular LAA exclusion, and epicardial clip occlusion should be preferred.^53^

## 8 CONTRAINDICATION FOR EPICARDIAL LAA CLIP CLOSURE

A relative contraindication for LAA clip exclusion derives from the potential negative effects on LV filling properties in patients with severe diastolic dysfunction. In such patients, increased LA compliance via increased volume is key to maintaining a higher reservoir function and thus lower postcapillary pressure. Acute amputation of the LAA, which is the most distensible part of the LA and accounts for 10%–20% of the left atrial volume,^54^ may cause increased pulmonary pressure and congestion symptoms. Nevertheless, we believe that due to the acute watertight feature of clip exclusion, epicardial LAA clip occlusion may have greater potential for diastolic impairment in selected high‐risk patients compared to transcatheter LAA occlusion devices. The latter work more like filters, and endothelialize gradually, leaving a residual distinct endoleak in up to 2/3 of the treated appendages.^55^

## 9 ANTICOAGULATION STRATEGY FOLLOWING LAA OCCLUSION

Selecting appropriate anticoagulation therapy following LAA occlusion remains a challenge. Under current AF anticoagulation guidelines, patients retain a class 1 indication for lifelong therapeutic anticoagulation based on CHA_2_DS_2_‐VASc risk factors, regardless of whether the LAA is present or not. There are no studies comparing epicardial LAA closure to OAC, so it cannot be considered a replacement treatment. At this time, a contraindication for anticoagulation seems to be the only strong indication for LAA exclusion. In patients with a history of previous uncontrolled life‐threatening bleeding, LAA occlusion followed by discontinuation of OAC/NOAC may be a reasonable approach, but requires a careful, tailored approach.

## 10 SURGICAL TECHNIQUES

**Figure 2:**
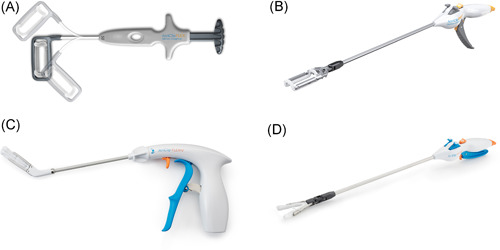
Different available LAA clip occlusion devices ((A) Atriclip Flex, (B) Pro2, (C) Flex V, (D) ProV). To be noted: Copyright is reserved to Atricure. LAA, left atrial appendage

Initially, surgical closure by means of stapling, amputation, ligation and/or direct sewing provided a high rate of incomplete exclusion, potentially increasing the risk of stroke due to the presence of a residual “pouch” with a thrombogenic effect.^9^, ^10^ In the past decade, surgical epicardial LAA occlusion by means of a preloaded nitinol clip (Gillinov‐Cosgrove LAA clip a.k.a. Atriclip®; AtriCure, Inc.) placed epicardially at the base of the LAA has proven to be a valuable tool with excellent results in terms of safe and durable LAA occlusion.^56^ Durability and efficacy results were confirmed at 3‐years follow‐up, in terms of complete occlusion and reduced incidence of stroke and cerebrovascular events in AF patients undergoing concomitant LAA occlusion.^57^, ^58^ The AtriClip is FDA‐approved device for epicardial LAA management. In recent years, several modifications have been made to the original Atriclip to facilitate minimally invasive use (Figure 2).

Ideally, the work up for surgical LAA management includes cardiac CT. This is important to rule out congenital anomalies such as persistent left superior vena cava and understand LAA morphology, while also providing noninvasive screening for possible LAA thrombus. As described above, TOE remains the gold standard to allow safe procedure planning and check for correct LAA clip deployment. TOE should be performed as close as possible to the LAA exclusion procedure, especially when a cardiac CT is not available or feasible (poor renal function, contrast allergy, impaired transportability). During the procedure, TOE allows for confirmation of freedom from thrombosis—of critical importance, since many patients will not be appropriately anticoagulated, if at all, at this stage—right before deployment, provides endoluminal feedback on a possible residual LAA stump, and helps to rule out possible residual leaks. The latter is less important, as no such findings have been reported since the introduction of the LAA clip.

After general anesthesia and using with selective lung ventilation, the TOE probe is inserted and defibrillation patches are applied. The patient is positioned in dorsal decubitus, with a slight lifting of the left side (we use inflatable balloons), just as one would prepare the right chest for minimally invasive mitral surgery, with the left arm left down.

**Figure 3:**
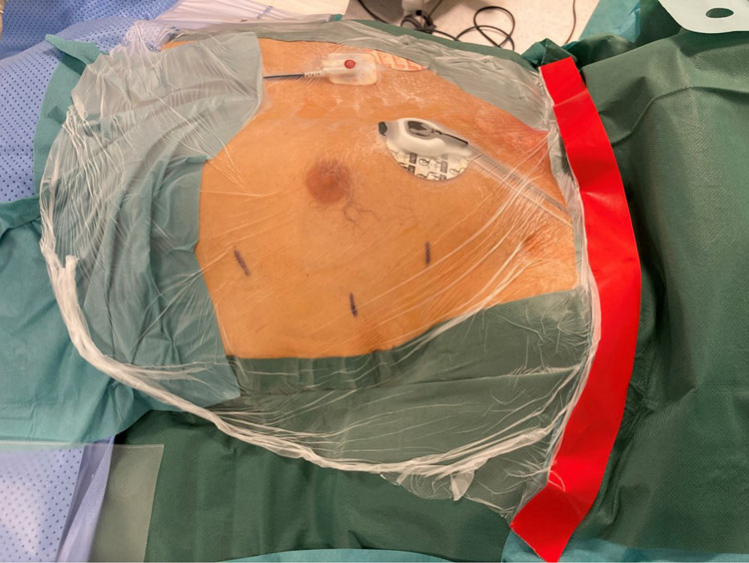
Patient and ports positioning

**Figure 4:**
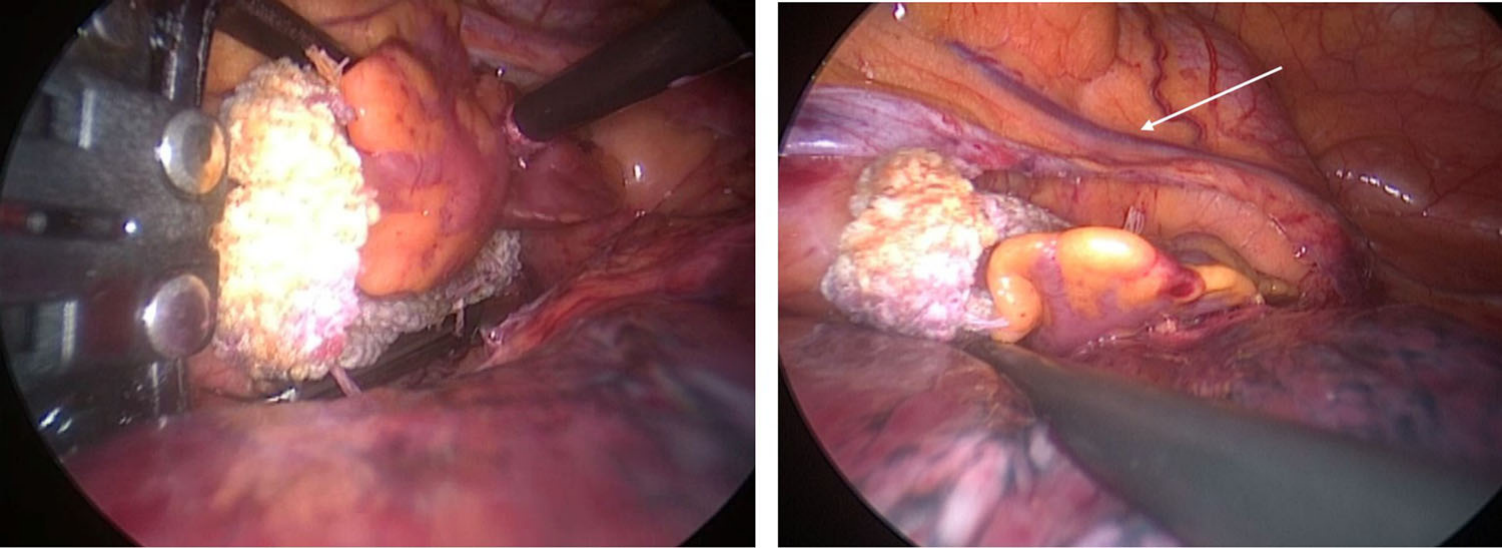
LAA occlusion with device positioning and release. Left: device positioning. Right: device release. LAA, left atrial appendage

Three ports are utilized: a camera port aligned with the midsternum (not considering the xyfoid), and two more anterior operating ports: a 5 mm port one intercostal space above and a 10–12 mm port circa 2 intercostal spaces below. (Figure 3) We use a 0° camera through a 10–12 mm viewport. After excluding the right lung from ventilation, aided by CO_2_ insufflation at 8–10 cm H_2_O, the pericardial sac is opened with endoscissors, cautery or harmonic forceps, parallel to and far from the phrenic pedicle. With normal mediastinal anatomy, retrophrenic pericardial access is preferred, though antephrenic pericardial incision can also provide good exposure, especially when the phrenic pedicle is located posteriorly. A single stay suture on the anterior side of the pericardiotomy may occasionally be used to enhance exposure. The base of the LAA is measured with the AtriClip sizer. A device of appropriate size is then selected and deployed through the inferior 12 mm port. Clip delivery is completely blunt and atraumatic (Figure 4).

Before releasing the device from the deployment tool, electrocardiogram might help rule out exceptionally rare instances of inadvertent coronary compression of the circumflex artery, which might be easily corrected by reopening and repositioning. Following clip placement, TOE is used to confirm complete exclusion, allowing assessment of any residual stump which may require a more proximal repositioning of the device. Although unreported so far with this technology, TOE control can confirm absence of residual leaks across the LAA clip in real‐time. Timing of such safety checks is critical; as most LAA clip placements are performed in total absence of anticoagulation, adjustments entailing reopening of the device can only be executed within one minute, as reopening of a LAA clip after longer periods of time might restore flow to an appendage containing thrombus. After LAA clip exclusion, the ports are closed leaving a pleural drainage in the lower port. The procedure lasts around 20 min in experienced hands and the patient is extubated directly post procedure and transferred to a stepdown ward.

## 11 SPECIAL CONSIDERATIONS

Due to the complete extracardiac nature of the procedure, no anticoagulation regimen is required. Therefore, LAA clip occlusion is highly suitable for those patients with relative and absolute contraindication to anticoagulant drugs, like those with ongoing bleeding complications.

Since the standard device (Figure 2) has a closed structure, positioning requires it to be placed around the LAA from its distal end, over the body, to the base of the structure, where it is closed and released. During this process, fresh thrombus within the LAA may be dislodged and embolize. Therefore, LAA thrombosis is a contraindication for LAA epicardial exclusion. Nevertheless, latest generation devices with a V shaped closing mechanism (Figure 2) may provide potential for LAA closure in those patients with refractory LAA thrombosis that is limited to the distal end of the appendage.^40^

In general, thoracoscopic LAA epicardial exclusion with an Atriclip device is a very safe and swift procedure.^59^ To maintain this very low risk threshold, we strongly recommend careful assessment of patients using imaging techniques as previously described (TOE, CT scan, and/or magnetic resonance imaging). From a surgical standpoint, we suggest staying >1 cm from the phrenic nerve, to avoid permanent or transient paralysis of the hemidiaphragm. It is very important to manage the LAA gently, avoiding grabbing the LAA as this tissue can be damaged easily. The device can be positioned very easily by pushing it open on top of the LAA while a‐traumatically “caressing” the LAA into the device frame with a peanut device.

**Figure 5:**
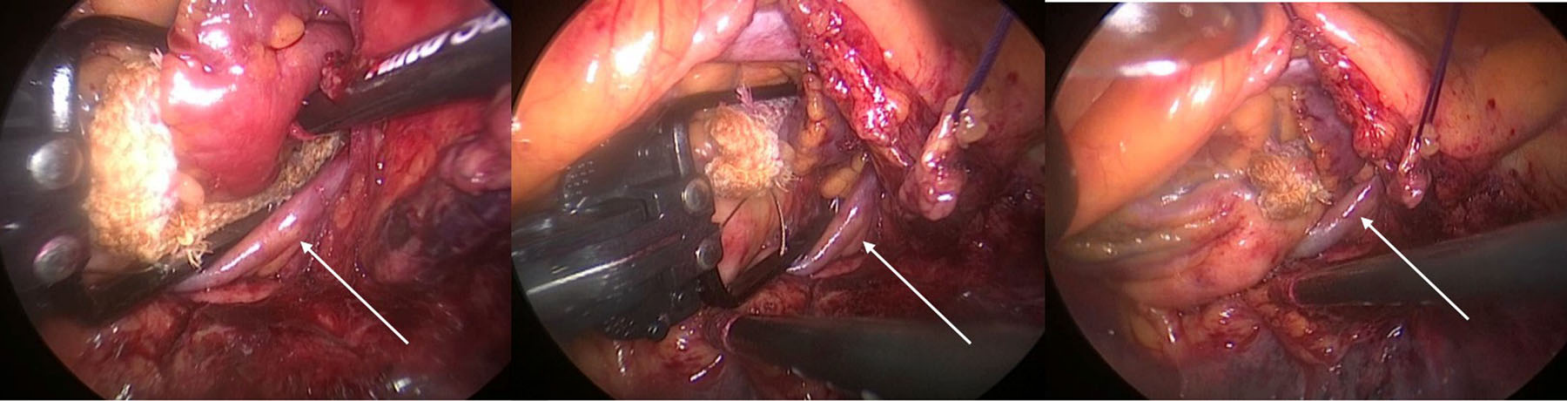
LAA occlusion in a patient with persistent superior vena cava (white arrow). Left: device positioning. Central: device closure. Right: device release. LAA, left atrial appendage

**Figure 6:**
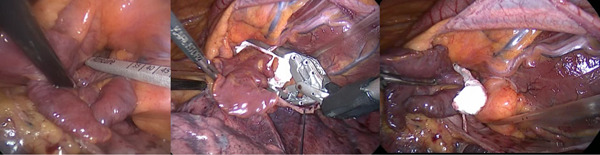
LAA occlusion in a patient with complete situs inversus dextrocardia. Left: device sizing. Central: device positioning. Right: device release. LAA, left atrial appendage

Finally, it is very important to understand how this procedure can be also performed in patients with different degree of cardiovascular abnormalities by experienced surgeons. Our experience shows how a complete LAA exclusion was obtained without additional risks in patients with a persistent left superior vena cava (Figure 5) and also in patients with dextrocardia with situs inversus (Figure 6).

## 12 CONCLUSIONS

There are several important clinical and theoretical advantages of epicardial LAA clip occlusion, including stroke risk reduction and improved rhythm outcome. Epicardial clip occlusion is reported to be feasible, safe, and very effective, however its role as a stand‐alone procedure remains to be established. Currently, isolated epicardial LAA clip closure is reserved for patients with a relative or strict contraindication for anticoagulation and/or antiplatelet therapy, and for those rare case of refractory automatic arrhythmias originating in the LAA. As a concomitant procedure, there is strong evidence that LAA closure can prevent stroke, although further research is needed and current guidelines do not include this recommendation yet.

Understanding of LAA morphology and pathology may help with planning epicardial LAA management.

Adding epicardial LAA clip to the technical armamentarium and openly discussing individual cases within an AF Heart Team is pivotal to developing a personalized approach and identifying the most appropriate LAA treatment for each individual patient.
